# Synergistic Effect of Essential Oils and Antifungal Agents in Fighting Resistant Clinical Isolates of *Candida auris*

**DOI:** 10.3390/pharmaceutics16070957

**Published:** 2024-07-19

**Authors:** Lorenza Cavallo, Francesca Menotti, Janira Roana, Cristina Costa, Fabio Longo, Claudia Pagano, Antonio Curtoni, Alessandro Bondi, Giuliana Banche, Valeria Allizond, Narcisa Mandras

**Affiliations:** 1 Department of Public Health and Pediatrics, University of Torino, 10126 Turin, Italy; lorenza.cavallo@unito.it (L.C.); francesca.menotti@unito.it (F.M.); janira.roana@unito.it (J.R.); cristina.costa@unito.it (C.C.); f.longo@unito.it (F.L.); claudia.pagano@edu.unito.it (C.P.); antonio.curtoni@unito.it (A.C.); alessandro.bondi@unito.it (A.B.); valeria.allizond@unito.it (V.A.); narcisa.mandras@unito.it (N.M.); 2 Azienda Ospedaliera Universitaria (A.O.U.) Città della Salute e della Scienza di Torino, 10126 Turin, Italy

**Keywords:** *Candida auris*, drug resistance, essential oils, antifungals, antifungal activity, antifungal agent combination, synergistic activity

## Abstract

Recently, a large number of nosocomial infections have been caused by an emerging pathogen that is rising as a worldwide issue in human health: *Candida auris*. This yeast is considered resistant to antifungals of the first-line therapies, and consequently it is related to morbidity and mortality. Therefore, the aim of this research was to determine the in vitro anti-*C. auris* activity against twenty-three resistant clinical strains of different essential oils (EOs), pure or in combination with traditional antifungal agents, mainly caspofungin, fluconazole, micafungin and 5-flucytosine. Broth dilution assay was performed to evaluate the fungistatic and fungicidal effectiveness of fifteen EOs towards all the *C. auris* isolates. The data demonstrated that EOs were able to prevent *C. auris* growth, with MIC values ranging from 0.03 to 1% for the efficacious EOs (thyme, cinnamon, geranium, clove bud, lemongrass and mentha of Pancalieri), whereas the MICs were >1% for the ineffective ones. Thereafter, the six most effective EOs were used to perform the checkerboard experiments by assaying simultaneously the activity of EOs and traditional antifungals towards two selected strains. The most promising synergic combinations towards *C. auris*, depending on the isolate, were those with micafungin and geranium, thyme, cinnamon, lemongrass or clove bud EOs, with fluconazole and mentha of Pancalieri EO, and with 5-flucytosine and mentha of Pancalieri EO. These EOs and their combinations with antifungal drugs may provide a useful therapeutic alternative that could reduce the dose of the individual components, limiting the overall side effects. These associations might be a prospective option for the future treatment of infections, thus helping to overcome the challenging issue of resistance in *C. auris*.

## 1. Introduction

In 2016, the Centre for Disease Control and Prevention (CDC) of the United States gave notice of the rise of a new multidrug-resistant *Candida* species: *C. auris* [1,2]. It was discovered in one Japanese and fifteen Korean patients in 2009, and has since been detected in more than 30 countries and linked to epidemics in health care institutions [3,4,5]. There have also been reported *C. auris*-related hospital outbreaks in Europe, and a consistent escalation in its recovery has been documented in Italy starting from 2020 [6]. New cases continue to emerge, and recent findings reveal that isolation rates of *C. auris* have practically doubled in the last few years [7]. *C. auris*—a yeast classified as Saccharomycetes belonging to phylum Ascomycota—causes severe illness, spreads quickly among patients in hospital and clinical settings, and significantly impacts their morbidity and mortality, as well as health care infrastructure and finance [8]. In fact, *C. auris* can cause a wide range of pathologies from superficial skin diseases to more severe, potentially fatal, infections like myocarditis, meningitis and osteomyelitis [9]. Clinical manifestations of *C. auris* are frequently non-specific and comparable to other forms of systemic infections, especially in critically ill patients in intensive care units [10]. 

This pathogen shows peculiar growth features, such as thermotolerance and osmotolerance, so it can grow at high temperatures (>40 °C) while being able to accept high salt concentrations (>10% NaCl), contributing to its long-term persistence and survival on either biotic or abiotic surfaces [11,12,13]. The virulence factors of *C. auris* include the ability to form large aggregates of pseudohyphal-like cells and to produce biofilm that together may facilitate the colonisation of the host’s epidermidis [14,15]. Furthermore, *C. auris* is a multidrug-resistant yeast which exhibits a variable susceptibility pattern to azoles, being resistant to fluconazole (FLU; MIC > 128 µg/mL), amphotericin B (AMB) and echinocandins [16,17]. As previously reported in the literature, especially after pre-exposure, this yeast shows nearly 100%, 30% and 5% resistance to FLU, AMB and echinocandins, respectively [18].

Considering that the number of accessible and currently used antifungal chemotherapeutics is extremely limited while the microbial resistance to these drugs is growing, the search for new therapeutic alternatives is crucial. The most promising choices compared to conventional treatment of fungal infections are plant products, such as essential oils (EOs) [19,20]. These have been employed for centuries for their therapeutic and aromatic properties. In recent years, in vitro studies have established that some of them are effective against common pathogenic fungi, including *Candida* spp., *Aspergillus* spp. and dermatophytes [21,22,23]. In particular, EOs are shown to be useful in treating infections caused by moulds and yeasts, displaying a good bioactivity and minimal toxicity when used in low quantities [24,25,26]. EOs can prevent the proliferation of these pathogens targeting multiple cell structures and/or functions, mainly by increasing membrane permeability, thus disrupting the cell membrane and causing the release of essential intracellular components, as well as interfering with cell metabolism and enzyme kinetics [27,28,29]. Additionally, the combination of EOs with antifungal drugs could be a further promising therapeutic approach in treating difficult drug-resistant infections [30]. According to various studies, EOs and antifungals can work in a synergic or additive manner, although useful outcomes can be achieved with combination therapy even when synergism is absent [31,32,33]. In this scenario, the use of an association of both antifungal and EO ensures the effectiveness of the treatment even without necessarily increasing the effects of either component. As a result, the overall therapeutic dose remains unchanged while the amount of each antifungal/EO required is reduced [33].

On these grounds, the aims of the present study were to evaluate the susceptibility pattern of twenty-three *C. auris* isolates to five antifungal drugs and fifteen EOs, and afterwards to estimate the promising interaction of the six most effective ones (thyme, cinnamon, geranium, clove bud, lemongrass and mentha of Pancalieri) with four traditional antifungals, specifically caspofungin (CAS), 5-flucytosine (5-FL), FLU and micafungin (MYC), in counteracting the growth of two selected *C. auris* clinical strains, isolated from a deep systemic infection and a cutaneous colonisation.

## 2. Materials and Methods

### 2.1. Yeast Strains

A total of twenty-three yeast strains were isolated from bilateral axillae and groin swabs, urinary and endovascular catheters, tracheal aspirates, bronchoalveolar lavages, sputum and blood cultures of hospitalised patients at Azienda Ospedaliera Universitaria (A.O.U.) Città della Salute e della Scienza di Torino—Molinette (Turin, Italy). At the Microbiology Laboratory of the same hospital, the isolated colonies were identified as *C. auris* by matrix-assisted laser desorption/ionisation–time-of-flight (MALDI-TOF) mass spectroscopy (Bruker Daltonics GmbH and Co., Brement, Germany), and all strains were preserved in cryovial tubes (Technical Service Consultants Ltd., Lancashire, UK) at −80 °C. To ensure the privacy of patients, each *C. auris* strain was named as Molinette (MOL) or Turin University Culture Collection (TUCC). 

Before the experiments, *C. auris* strains were streaked on Sabouraud dextrose agar (SAB-A; Biokar diagnostics, Beauvais, France) and incubated for 24 h at 35 °C to ensure purity.

### 2.2. Antifungal Drugs and Essential Oils

The antifungal drug powders (≥98% purity by HPLC) CAS, FLU, 5-FL and MYC were purchased from Merck Life Science S.r.l. (Milan, Italy). To reach a concentration of 1000/1 µg/mL, CAS, 5-FL and MYC powders were dissolved in 100% dimethylsulfoxide (DMSO; Merck Life Science S.r.l.), whereas FLU was solubilized in sterile purified water (Otec, Lyon, France) and stored at −80 °C until use. These standard antifungal drugs were used to determine the susceptible/resistant profile of the clinical selected yeasts.

The fifteen EOs used in the present research were as follows. *Cinnamomum zeylanicum* (cinnamon), *Citrus bergamia* (bergamot), *Citrus limon* (lemon), *Coffea arabica* (Arabica coffee), *Commiphora myrrha* (myrrh) *Commiphora wildii* (Namibian myrrh), *Piper nigrum* (black pepper), *Thymus zygis* (thyme) and *Zingiber officinali roscoe* (ginger) EOs were kindly provided by MANE (www.mane.com). *Lavandula officinalis* (lavender)*, Malaeuca alternifolia* (tea tree), *Syzygium aromaticum* (clove bud)*, Paerlargonium graveolens* (geranium) and *Cymbopogon nardus* (lemongrass) EOs were supplied by Primavera, Flora s.r.l. (Lorenzana, Pisa, Italy). Finally, *Mentha × piperita* var. *officinalis* (mentha of Pancalieri) was purchased from Erbe Aromatiche Essenzialmenta (Pancalieri, Turin, Italy).

### 2.3. Antimicrobial Assays

#### 2.3.1. European Commission-In Vitro Diagnostic Broth Dilution Test

The European Commission in vitro diagnostic (EC-IVD) broth microdilution method with Micronaut-AM plate (Bruker Daltonics GmbH and Co.) was used to screen the susceptibility pattern of all *C. auris* strains against AMB (range 16–0.03 µg/mL), anidulafungin (ANF; range 8–0.016 µg/mL), CAS (range 8–0.016 µg/mL), 5-FL (range 32–0.06 µg/mL), FLU (range 128–0.25 µg/mL), itraconazole (ITZ; range 4–0.03 µg/mL), MYC (range 8–0.016 µg/mL), posaconazole (POS; range 8–0.008 µg/mL) and voriconazole (VOR; range 8–0.008 µg/mL). A working solution of 1 × 10^3^ colony forming units (CFU)/mL was prepared by firstly suspending 1:20 the 0.5 McFarland Standard yeast suspension in 0.9% sterile NaCl and further diluting 1:50 the resulting suspension in Micronaut-RPMI-1640 medium with M 3-(N-morpholino) propane sulfonic acid (MOPS) and glucose. Subsequently, 100 µL of the 1 × 10^3^ CFU/mL was transferred in each well of the Micronaut-AM plate and incubated at 37 °C for 24 h. After the incubation time, a visual evaluation was made; the fungal growth was indicated by a colour change from blue to pink mediated by the AST indicator supplemented to the test medium.

#### 2.3.2. Handmade Broth Dilution Test

The handmade broth microdilution assay was used to evaluate the antimicrobial activity of both antifungals and EOs according to the European Committee on Antimicrobial Susceptibility Testing (EUCAST) guidelines, specifically the EUCAST method for susceptibility testing of yeasts (v. 7.3.2) (https://www.eucast.org/astoffungi/previous_versions_of_documents, accessed on 15 May 2020).

Before the experiments, all the *C. auris* isolates were cultured overnight at 35 °C by placing a bead of the cryovials in Sabouraud dextrose broth (SAB-B; Biokar diagnostics). Thereafter, the inoculum of each *C. auris* strain was reached by centrifuging at 4000× *g* 10 min the overnight culture on SAB-B and by dissolving the pellet in 0.9% sterile NaCl solution, yielding a 0.5 McFarland standard (5 × 10^6^ CFU/mL) yeast suspension. A working inoculum of 0.5–2.5 × 10^5^ CFU/mL was obtained by a 1:10 dilution in RPMI-1640 medium (Sigma-Aldrich, St. Louis, MO, USA) enriched with 2% glucose (Sigma-Aldrich) and buffered to pH 7.0 with 0.165 MOPS (Amresco LLC, Solon, OH, USA), from now reported as RPMI-1640 medium with MOPS for brevity.

The minimum inhibitory concentration (MIC) determination was carried out in RPMI-1640 with MOPS, using 96-well microtiter plates. Firstly, stock of both EOs and antifungals was prepared as follows. The EO standard solutions were produced in DMSO (1:2.5 *v*/*v*), then diluted (1:20 *v*/*v*) to reach a final concentration of 2% (*v*/*v*) in RPMI-1640 medium with MOPS, and supplemented with a 0.001% *v*/*v* of Tween 80 (Sigma-Aldrich) to enhance EO solubility without inhibiting yeast growth. In parallel, the 1000/1 µg/mL drug stocks were used to prepare the final concentration of 4/1 µg/mL for CAS and MYC, and of 2/1 µg/mL for 5-FL, whereas FLU was used directly at 1000/1 µg/mL to set up the microtiter plate suspensions. Secondly, the two-fold serial dilutions of EOs (range 1–0.001% *v*/*v*), CAS (range 4–0.001 µg/mL), MYC (range 4–0.001 µg/mL), FLU (range 512–0.25 µg/mL) and 5-FL (range 2–0.001 µg/mL) were performed and 100 µL was added in each well of the microtiter plates. Finally, 100 µL of the yeast inoculum suspension (~10^5^ cells/mL) was placed in each well. The microtiter plates were then incubated for 24 h at 35 °C. Sterile RPMI-1640 medium with MOPS—incubated under the same conditions—was used as a negative control, while the positive growth control was set up with medium inoculated with the tested *C. auris*. All the experiments were carried out in duplicate and repeated at least three times. The microdilution plates were read using a microtiter plate reader (VICTOR3TM, PerkinElmer, Boston, MA, USA) with a wavelength of 490 nm. The MIC was defined as the lowest concentration of antifungal or EO which inhibited 50% or more of the yeast proliferation compared to the positive growth control.

To ascertain the minimal fungicidal concentration (MFC), 10 μL from the wells starting from MIC and onwards was subcultured onto SAB-A plates. Following 24 h of incubation at 35 °C, MFC was defined as the lowest concentration of drug or EO that killed 99.9% of the inoculum as no growth on the subcultures was revealed.

#### 2.3.3. Agar Disc Diffusion Assay

The agar disc diffusion test was used to investigate the antimicrobial activity of the six most effective EOs: cinnamon, clove bud, geranium, lemongrass, mentha of Pancalieri and thyme, and four antifungal drugs: CAS, FLU, 5-FL and MYC, on two selected *C. auris* strains (details in Section 2.4). According to the method described by Fernandes et al. [16], a sterile swab was used to spread a previously prepared yeast suspension of ~1 × 10^8^ CFUs/mL, equivalent to 6 McFarland standard density, on SAB-A plates. Afterwards, discs loaded with 25 µL of 100% *v*/*v*, 75% *v*/*v* and 25% *v*/*v* of each EO, or with 25 µL of the 1000/1 of FLU and 5/1 µg/mL of CAS, MYC and 5-FL, were placed on the agar surface. All the plates were subsequently incubated for 24 h at 35 °C. Finally, the diameter of the area around the disc where the yeast growth stopped was measured in millimetres (mm): when the halo was ≤8 mm the *C. auris* was deemed resistant, whereas when it was >8 mm the strain was considered susceptible [16]. The results of the diameters of the inhibition halos were reported as mean ± standard deviation (SD) of three different experiments.

### 2.4. Checkerboard Assays and Assessment of Fractional Inhibitory Concentration Index

The interaction between the antifungal drugs and the six EOs with the lowest MICs was investigated in vitro using a modified checkerboard assay as previously detailed [33,34]. *C. auris* MOL 10—from deep systemic infection—and *C. auris* MOL 11—from cutaneous colonisation—were employed as challenge microorganisms. The following EOs were tested: cinnamon, clove bud, geranium, lemongrass, mentha of Pancalieri and thyme, whereas the antifungal drugs used were CAS, FLU, 5-FL and MYC. 

Based on their MIC values, serial two-fold dilutions of the antifungal drugs and EOs were carried out, ranging from several dilutions below the MIC to 2× MIC, and were prepared in a two-dimensional checkerboard. In a 96-well microtiter plate, binary combinations were mixed together. Following microplate preparation, the fungal inoculum (0.5–2.5 × 10^5^ CFU/mL) was transferred into each well, and the plates were then incubated for 24 h at 35 °C before being measured at 490 nm.

**Figure 1:**
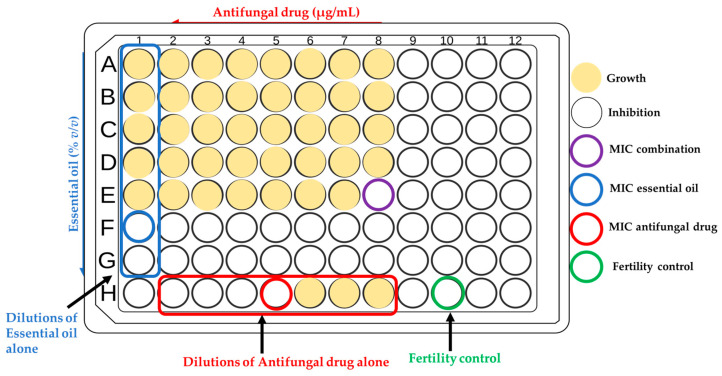
Representative image of the 96-well plate reporting the dilution of both antifungals and EOs, and the resulting interpretation of the checkerboard test.

To accurately understand the results, one column was set aside to be used for the EO dilution control alone, while another was designated only for the drug dilution control. All the experiments were carried out in duplicate and repeated at least three times. Figure 1 presents an example of the 96-well plate preparation reporting the dilutions of the checkerboard experiment to evaluate the interaction between antifungals and EOs.

The following formula was used to determine the fractional inhibitory concentration index (FICI):

$$FIC\;of\;Essential\;Oil=\frac{MIC\;of\;Essential\;Oil\;in\;Combination}{MIC\;of\;Essential\;Oil\;Alone}$$

$$FIC\;of\;Antifungal=\frac{MIC\;of\;Antifungal\;in\;Combination}{MIC\;of\;Antifungal\;Alone}$$

FICI = FIC of Essential Oil + FIC of Antifungal

The FICIs were interpreted as previously described by Parker et al. [33]. FICI ≤ 0.5, synergy; 0.5 < FICI < 1, additivity; 1 ≤ FICI < 4, indifference; FICI ≥ 4, antagonism.

## 3. Results and Discussion

### 3.1. C. auris Isolates and Antifungal Susceptibility Patterns

Twenty-three yeast strains were isolated from different clinical specimens, as follows: bilateral axillae and groin swabs (n = 13), urinary catheter (n = 3), blood cultures (n = 3), endovascular catheter (n = 1), tracheal aspirates (n = 1), bronchoalveolar lavages (n = 1) and sputum (n = 1), and identified as *C. auris*. Table 1 summarises the susceptibility pattern of these clinical isolates determined by Micronaut-AM. Briefly, all the tested strains were resistant to FLU, reporting MIC values > 128 μg/mL, whereas for VOR and ITZ they displayed MICs ranging from 0.0078 to 2 μg/mL and from 0.06 to >4 μg/mL, respectively. The most effective azole was POS, with MIC distribution between 0.0078 and 0.25 μg/mL. Pertaining to echinocandins, the obtained MIC values were lower than those of azoles, ranging from 0.03 to 0.5 μg/mL. Only for *C. auris* TUCC 306 was a MIC of >8 μg/mL recorded for CAS, indicating a resistant strain. The AMB susceptibility profile showed a MIC distribution between 0.25 and 2 μg/mL. Finally, the most effective antifungal agent was 5-FL, as MICs were less than 0.06 μg/mL in most of the isolates (19/23; 82.61%).

Literature data on the sensitivity pattern of *C. auris* clinical strains confirmed their resistance to numerous antifungal agents [4,16,17,18,35,36,37,38,39,40]. In a recent study, about 1000 *C. auris*, isolated from various clinical specimens, were assayed in vitro for susceptibility to conventional antifungals, and a high percentage of resistance to FLU was demonstrated, in agreement with our data. Additionally, other antifungals displaying a variable pattern, such as AMB, ITZ and CAS, to which most of the strains were susceptible, confirmed the results reported here [35]. The Jacobs research group [37] observed a wide resistance to FLU as well as a susceptibility to echinocandins and AMB in *C. auris*, data similar to ours. Conversely, they registered a 5-FL resistant profile in some strains. More recently, ten isolates of *C. auris* were investigated for their susceptibility to six of the most clinically used antifungals by microdilution assays. These underlined, in accordance with our data, higher MICs for FLU and AMB, and lower MIC values for 5-FL [17], whereas a systematic review on different compounds and antimicrobials revealed that only a few of them were able to counteract *C. auris* growth and/or biofilm formation [36]. 

Notably, of all the *C. auris* isolates, two strains—representative of two different sites of collection, deep and superficial—specifically MOL 10 (blood culture) and MOL 11 (tissue swab), were selected and subjected to further experiments. In parallel, the MIC profiles were determined by the handmade broth dilution assay using an inoculum of *C. auris* at 10^5^ yeast/mL as indicated in EUCAST guidelines. The MIC results were as follows: FLU > 128 μg/mL, MYC 1 μg/mL, CAS 0.125 μg/mL and 5-FL 0.125 μg/mL for *C. auris* MOL 10, whereas FLU > 128 μg/mL, MYC 2 μg/mL, CAS 0.25 μg/mL and 5-FL 0.5 μg/mL for *C. auris* MOL 11. These results are supported by some authors who underlined that the different techniques used for the susceptibility pattern determination, broth dilution methodologies and E-test, led to some discordances in the outcomes [37]. 

### 3.2. C. auris Essential Oil Susceptibility Patterns

The whole chemical composition of EOs, as well as their individual components, confer on them antimicrobial properties, and in some cases, the EO fractions are more effective than the entire EO against fungi [21,22,23,39,41]. Table 2 details the composition of EOs as defined through the producer’s analysis and results.

Literature data corroborate the effect of EOs on different moulds (*Aspergillus* spp. and dermatophytes) and yeasts, such as *Candida* spp. [21,22,23,31,32]. Likewise, our research group previously demonstrated the anti-*Aspergillus* spp. efficiency of eleven EOs, especially those of lemongrass, clove bud and geranium [22]. Moreover, the efficacy of both EOs and their components against *Candida non-albicans* species was previously underlined [21,42]. Therefore, to achieve this goal against *C. auris* as well, the MIC evaluation of all the EOs was determined by the broth dilution method—as previously described—with respect to the twenty-three *C. auris* clinical strains [21,43].

EOs exhibit a limited time of action due to their volatility, but the presence of individual volatile compounds—terpenoids and non-terpenoids—containing the hydroxyl radical (^•^OH) displays antioxidant activity that enhances the antimicrobial properties of the EOs [44]. As detailed in Table 3, the least effective EOs against all the *C. auris* isolates were lavender, black pepper, tea tree, coffee, bergamot, ginger, lemon, myrrh and myrrh of Namibia, displaying MICs ranging from 0.5 to >1% *v*/*v*. A slight activity was achieved for mentha of Pancalieri (MICs 0.125–1% *v*/*v*). On the other hand, regarding geranium, clove bud, lemongrass and cinnamon, the antifungal activity was higher, with MIC values between 0.03 and 0.125% *v*/*v*. The most effective EO was thyme, demonstrating MICs from 0.015 to 0.06% *v*/*v* for all the assayed *C. auris* clinical strains. Table 4 reports the MFC values, and a similar activity pattern to those of MICs was noted.

In a study, the components of different EOs, specifically eugenol, methyleugenol, carvacrol and thymol, were assayed by dilution method for their anti-*C. auris* efficacy, revealing that carvacrol and thymol displayed the lower MIC values [39]. Parker et al. [33] assayed numerous EOs with regard to *C. auris*, and in full agreement with our data, cinnamon leaf, clove bud, geranium, lemongrass, peppermint and tea tree demonstrated strong activity in stopping *C. auris* growth. Di Vito et al. and Rosato et al. [17,40,41] evaluated the anti-*C. auris* activity of cinnamon EO or its components, and highlighted MICs of 0.06% *v*/*v*, in accordance with our results. The data here obtained revealed that not only cinnamon but also thyme and clove bud were the most effective EOs towards almost all the *C. auris* clinical strains, and this could be explained by the main components of these EOs, which are thymol and eugenol, respectively (Table 2). Indeed, these molecules display a pronounced antifungal activity, in accordance with previous literature findings, and further confirming that the EO composition influences its outcome in terms of antimicrobial properties [17,33,39,40,41]. More recently, numerous EOs were tested against one strain of *C. auris*, and the most active EO was that extracted from *Lippia alba*, carvone–limonene chemotype [45]. 

In a previous study, the authors assayed the action of EOs and commercial terpenes against seven *C. auris* strains susceptible or resistant to FLU or AMB: the results underlined high values of MICs (as µg/mL) independently from the used compound [46]. Moreover, myrtenol was tested against a strain of *K. pneumoniae* and one of *C. auris*, and antimicrobial activity was revealed only for the latter [47]. Conversely, Kim et al. [48] did not detect an inhibitory effect towards *C. auris*, or other *Candida* spp. isolates, in the presence of 6-shogaol (ginger).

**Figure 2:**
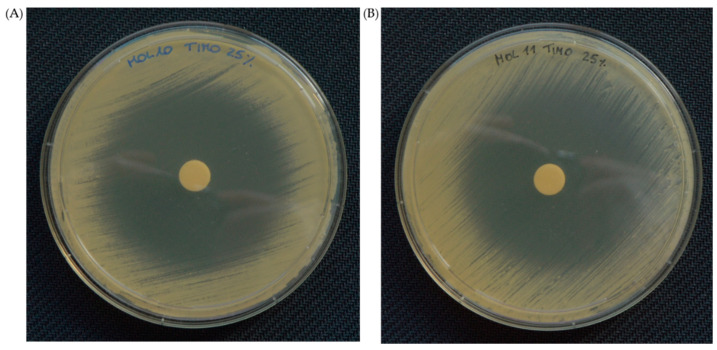
Representative images of the inhibition halo in the presence of thyme EO at the lowest concentration (25%), towards *C. auris* MOL 10 (**A**) and MOL 11 (**B**).

**Figure 3:**
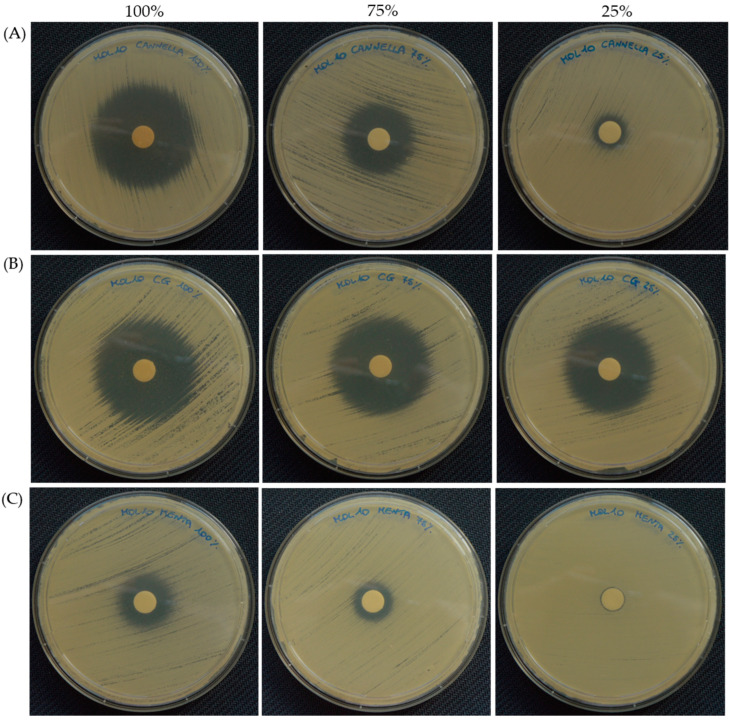
Representative images of the inhibition halo in the presence of cinnamon (**A**), clove bud (**B**) and mentha of Pancalieri (**C**) EOs at diminishing concentrations, specifically 100%, 75% and 25%, towards *C. auris* MOL 10.

**Figure 4:**
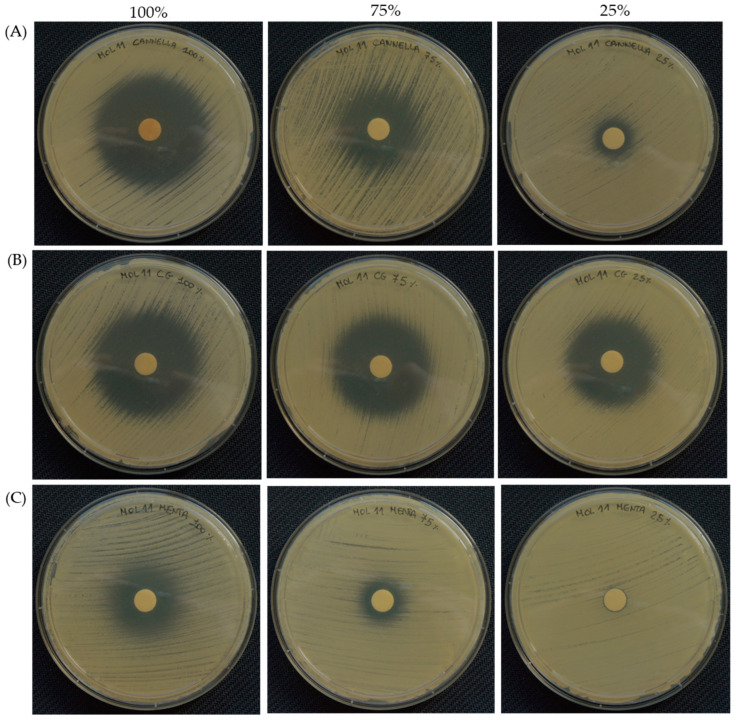
Representative images of the inhibition halo in the presence of cinnamon (**A**), clove bud (**B**) and mentha of Pancalieri (**C**) EOs at diminishing concentrations, specifically 100%, 75% and 25%, towards *C. auris* MOL 11.

The agar disc diffusion test was used to further assess the activity of both the antifungals—assayed at 1000/1 and 5/1 µg/mL—and the six most effective EOs—used at 100%, 75% or 25%—towards the two selected *C. auris* strains (MOL 10 and MOL 11). Regarding the antifungals, 5-FL (~39 mm) displayed a larger inhibition halo compared to echinocandins (MYC~24 mm and CAS~14 mm), while FLU was confirmed to be ineffective—with no inhibition halo—on *C. auris*, both MOL 10 or MOL 11 (Table 5). The results shown in Table 6 also demonstrated the variability of the inhibition halo diameter depending on the EO assayed and/or its concentration, with an efficacy akin to that of the handmade broth dilution data. In particular, all the EOs’ diameters were greater than 8 mm, and therefore both clinical strains displayed a susceptible profile. Once again, thyme was the most active EO, showing an inhibition halo almost equal to the diameter of the Petri dish (Figure 2), followed by clove bud, lemongrass, cinnamon and geranium EOs. Remarkably, the halo diameter of clove bud EO did not vary with the increase of its concentration in the disc, and therefore a minor amount of oil might be used for therapeutic purposes. However, for mentha of Pancalieri EO, for which the broth dilution revealed higher MIC values, the inhibition halo was the smallest for both *C. auris* strains. This could be explained by the lower release capability of this EO compared to the others. Figure 3 and Figure 4 depict representative pictures of the inhibition halo against *C. auris* MOL 10 and MOL 11 in the presence of the EOs, respectively.

Notably, few reports used both techniques—broth dilution and agar disc diffusion—to assess the anti-*C. auris* efficacy of EOs as we did in our experiments. Among them, Fernandes et al. [16] determined the anti-*C. auris* activity of different EOs, specifically thyme, tea tree, cajeput and niaouli, by using both methods. These authors demonstrated a lower MIC for tea tree EO (0.78% *v*/*v*) compared to our results (1% *v*/*v*), and a higher MIC for thyme EO (1.56% versus ≤ 0.06% *v*/*v*). The disc diffusion assay demonstrated an inhibition halo of ~20 mm for either tea tree or thyme EO, while in our study, a greater diameter of inhibition was registered for the latter. Analogously, *Cinnamomum zeylanicum* was used to estimate the inhibition of the growth of *C. auris*: a halo of ~73 mm and a MIC value of 0.06% *v*/*v* were observed, in accordance with our data [29]. 

### 3.3. Checkerboard Experiments on C. auris and EOs/Antifungals

Following the broth dilution experiments, the six most effective EOs were employed for the checkerboard assays—towards MOL 10 (Table 7) and MOL 11 (Table 8) clinical isolates—by testing four traditional antifungal drugs: FLU, MYC, CAS and 5-FL. The data were interpreted according to Parker et al. [33]. An antagonistic effect was revealed for the CAS and thyme combination towards *C. auris* MOL 10, while no antagonism was obtained against MOL 11. Conversely, the vast majority of the EOs showed an additive or indifferent activity when combined with antifungals for both strains. In particular, for *C. auris* MOL 10, an additive effect was revealed for twelve combinations, with FICIs ranging from 0.75 to 0.99; and the indifferent profile was obtained for eight associations, with FICIs varying between 1.06 and 2.5 (Table 7). For *C. auris* MOL 11, ten additive (FICIs 0.97–0.99) and nine indifferent (FICIs 1.5–2) combinations were observed (Table 8). Interestingly, the synergistic effects (FICIs 0.5) were highlighted for both strains: pertaining to MOL 10, the combinations between MYC and geranium, thyme or cinnamon EOs; regarding MOL 11, the associations between FLU and mentha of Pancalieri, MYC and lemongrass, clove bud or cinnamon, and 5-FLU and mentha of Pancalieri. Notably, when MYC and cinnamon EO were assayed alone, the MIC values obtained were fairly high (1–2 µg/mL for MYC and 0.25% *v*/*v* for cinnamon), whereas for both MOL 10 (blood culture) and MOL 11 (tissue swab), the checkerboard test revealed the synergistic effect of their combination. This might be explained by the fact that in cinnamon EO, eugenol is the most abundant compound. Indeed, this EO component is capable of damaging the cell wall and disrupting the cytoplasmic membrane of *Candida* spp. which, in turn, could allow better access of MYC to its target [49,50,51]. 

Numerous articles evaluated the combined activity of antifungals, mainly azoles, echinocandins, AMB and 5-FL, in their in vitro ability to counteract *C. auris* multiplication [35,37,38,52,53,54]. Recently, O’Brian and colleagues [35] estimated that different antifungal drugs used together were able to reduce *C. auris* growth, and they also revealed that the most effective combination was that of VOR and 5-FL. Analogously, data from Jacobs et al. [37] underlined that pan-resistant strains of *C. auris* were affected in their proliferation by using combined antifungals (i.e., AMB and CAS). Furthermore, towards *C. auris* strains, a synergistic activity was demonstrated by the association of MYC and VOR, but an indifferent interaction was obtained for FLU and CAS or MYC [38]. In this context, colistin was employed with echinocandins to counteract *C. auris* multiplication, thus revealing that, as expected, this antibiotic alone did not affect the yeast proliferation, whereas in combination with CAS a synergic activity was observed [52].

Until now, only a few articles have reported the in vitro evaluation of the combined effect of EOs and antifungal drugs. To the best of our knowledge, this is the first report that includes a relevant number of European isolates of *C. auris* harvested from both superficial and deep infection sites, and that evaluates, for all the strains, the antifungal activity of nine traditional drugs and fifteen EOs, with different methodological approaches. Remarkably, the checkerboard assay was also set up by using two representative clinical isolates and numerous combinations of EOs/antifungals. Recently, the associated activity of cinnamon EO and FLU or MYC was evaluated, and an additive profile was recorded, as demonstrated for *C. auris* MOL 10 in our results [17,40]. Notably, from the results reported here, when cinnamon EO was associated with CAS, a synergic effect was further noted for both of the strains isolated from the two different sites, either deep (MOL 10) or shallow (MOL 11). Parker et al. [33] assessed the association of antifungals and EOs in fighting *C. auris* growth in vitro: a synergistic activity was registered only for AMB and clove bud, whereas, in our data, it was additionally highlighted for MYC with either geranium or thyme EOs for *C. auris* MOL 10, or with lemongrass, cinnamon and clove bud EOs for *C. auris* MOL 11. Moreover, the same authors observed that FLU and 5-FL were generally synergic (es. clove bud) or additive (es. cinnamon)—in accordance with our results—but depending on the EOs used for the combination [33]. Maione and colleagues [47] evaluated the association of myrtenol with CAS or meropenem towards *C. auris* and *K. pneumoniae*, respectively, and a synergic effect was obtained. Our checkerboard experiments confirmed the MIC results of EOs, and in fact, a synergism was highlighted for the combination of MYC with thyme and/or cinnamon and/or clove buds. Once again, the results obtained with these EOs, abundant in eugenol or thymol, further demonstrated, as stated above, that the composition of EOs is crucial for their activity against the *C. auris* isolates either alone or in combination with antifungals. Particularly, regarding MOL 11—the strain harvested from the skin—a synergistic activity was also noted for mentha of Pancalieri EO in combination with FLU or 5-FL, corroborating the fact that even if the EO or the drug alone might be ineffective, their association can enhance the anti-*C. auris* outcome. Accordingly, Di Vito et al. [41] assayed the combination of FLU and *Cinnamomum zeylanicum* EO fraction, and an additive antifungal activity was noted on *C. auris*. Finally, Shaban et al. [39] determined the most proper association of carvacrol, a compound of EOs, with antifungals to achieve a synergism, and this was obtained in its combination with AMB or nystatin.

Since similar concentrations of novel antimicrobial compounds, such as EOs or metal ions, are active against both eukaryotic cells and microorganisms, the tuning of their optimal quantity—necessary to reach antifungal activity without hampering human cell viability—is highly desired [25,55]. Adukwu et al. [26] revealed that lemongrass EO at 0.126% *v*/*v* was not toxic for human dermal fibroblasts. Additionally, in a previous study, our research group demonstrated that, when EOs are blended in a polymer-based three-dimensional scaffold, an amount of either EO (cinnamon) or its compound (eugenol) lower than 10% *v*/*v* did not impaired the viability of eukaryotic cells, specifically human sarcoma osteogenic-2 cells [25]. Similarly, the loading of thyme EO into nanoparticles, made of poly(3-hydroxybutyrate-co-3-hydroxyvalerate), did not affect the survival of VERO cells when its release was at about 2% [56]. Therefore, we can speculate that all the EOs here, used at a maximum concentration of 1% *v*/*v*, should be considered as non-toxic for human cells.

## 4. Conclusions

As known, the rate of resistance emergence to antimicrobial agents is slower in fungi compared to bacteria. However, *C. auris* arose as a critical pathogen in health care institutions, as it is resistant to fluconazole while exhibiting a variable susceptibility pattern to the other first-line drugs. Therefore, it is necessary to explore new therapeutic options, such as EOs or their combination with antifungals, to successfully overcome these challenging infections. In the present study, we assess—through different methodologies—the pattern of susceptibility/resistance of numerous European strains of *C. auris*, isolated from deep and superficial infections, towards both traditional antifungals and EOs. The in vitro results here reported indicate that EOs are able to inhibit the growth of fluconazole-resistant *C. auris* clinical isolates. In particular, the most effective ones are thyme, cinnamon, geranium, clove bud, lemongrass and mentha of Pancalieri. Notably, the checkerboard findings, achieved by exploring a relevant number of EO and antifungal associations, demonstrate—to various extents—that the combination of an antifungal drug with EOs can enhance the anti-*C. auris* activity, reaching a synergic or additive effect. Hence, we confirm that EOs display antifungal features alone, and might also boost the effectiveness of antifungals which would otherwise be impaired in their activity towards resistant strains. In this context, the use of an association could lead to a reduction in the dose of the individual components, thus limiting the overall side effects.

Additionally, an interesting approach to overcome the limitations of EOs related to their volatile nature, lipophilicity and possible toxicity at high concentration would be the development of nanovehicles encapsulated with both antifungals and EOs to counteract the cutaneous colonisation of *C. auris*, to prevent its shift to the deeper layers and subsequent systemic infections. Moreover, this might also be a promising strategy for the targeted delivery of drugs for deep mycosis counteraction, implying a different posology and limiting systemic adverse reactions. Therefore, these associations might also be used in hospital and clinical settings, only after ex vivo and in vivo evaluations.

## Figures and Tables

**Table 1:** Minimum inhibitory concentration (MIC) of all the antifungal drugs towards *C. auris* clinical isolates, reported as μg/mL by Micronaut-AM.

Strains	FLU	ITZ	VOR	POS	ANF	MYC	CAS	AMB	5-FL
MOL 1	>128	0.125	1	0.06	0.06	0.06	0.125	1	≤0.06
MOL 2	>128	1	1	0.125	0.125	0.06	0.125	1	0.125
MOL 3	>128	0.25	1	0.06	0.03	0.03	0.06	1	≤0.06
MOL 4	>128	0.25	0.5	0.06	0.03	0.03	0.06	1	≤0.06
MOL 5	>128	0.25	0.0078	0.0078	0.06	0.03	0.06	0.25	0.06
MOL 6	>128	0.125	0.25	0.03	0.03	0.03	0.125	0.5	≤0.06
MOL 7	>128	1	1	0.25	0.125	0.06	0.125	1	≤0.06
MOL 8	>128	0.125	0.5	0.03	0.016	0.016	0.06	0.125	≤0.06
MOL 9	>128	1	0.5	0.03	0.0125	0.03	0.125	1	≤0.06
MOL 10	>128	0.25	0.5	0.06	0.06	0.06	0.125	1	≤0.06
MOL 11	>128	0.125	0.5	0.06	0.25	0.125	0.25	0.5	≤0.06
MOL 12	>128	1	1	0.06	0.06	0.06	0.125	0.25	≤0.06
MOL 13	>128	0.06	0.5	0.03	0.125	0.06	0.125	0.25	≤0.06
MOL 14	>128	0.125	0.5	0.06	0.125	0.125	0.125	0.25	≤0.06
TUCC 306	>128	>4	2	0.25	0.5	0.25	>8	2	≤0.06
TUCC 307	>128	2	1	0.06	0.06	0.06	0.125	0.5	0.5
TUCC 308	>128	0.125	0.5	0.06	0.03	0.03	0.125	1	1
TUCC 309	>128	0.125	0.5	0.06	0.06	0.03	0.125	1	≤0.06
TUCC 353	>128	0.5	0.5	0.06	0.125	0.06	0.125	1	≤0.06
TUCC 355	>128	0.06	0.5	0.03	0.06	0.06	0.125	0.25	≤0.06
TUCC 356	>128	0.06	0.5	0.03	0.06	0.03	0.125	0.25	≤0.06
TUCC 358	>128	0.06	0.5	0.03	0.06	0.03	0.125	0.5	≤0.06
TUCC 359	>128	0.06	0.5	0.03	0.06	0.03	0.06	0.5	≤0.06

Abbreviations: 5-FL: 5-flucytosin; AMB: amphotericin B; ANF: anidulafungin; CAS: caspofungin; FLU: fluconazole; ITZ: itraconazole; MOL: Molinette; MYC: micafungin; POS: posaconazole; TUCC: Turin University Culture Collection; VOR: voriconazole.

**Table 2:** Scientific and common names, and chemical composition of the EOs used in the present study.

Scientific Name	Common Name	Main Components
*Cinnamomum zeylanicum* Blume	Cinnamon	83.35% eugenol; 3.68% benzyl benzoate; 2.57% trans β-caryophyllene; 1.88% eugenil acetate; 1.56% cinnamaldehyde
*Citrus bergamia* Risso & Poit.	Bergamot	98% bergamot essential oil; 0.8% citral; 0.08% geraniol; 0.04% citronellal; 0.02% Carvone
*Citrus limon* L.	Lemon	69.25% limonene; 11.37% pinene; 7.86% γ-terpinen; 1.98% sabinene; 1.75% α-pinene
*Coffea arabica* L.	Arabica Coffee	99.9% Arabica coffee pure essential oil; 0.1% isoeugenol
*Commiphora myrrha* Jacq.	Myrrh	80% Myrrh essential oil; 5% (−)-germacrene D; 1% trans β-carophyllene; 1% β-ocimene; 1% farnesol
*Commiphora wildii* Merxm.	Namibian Myrrh	80% α-pinene; 10% β-pinene; 1% paracymene; 1% 4-terpinenol; 1% sabinene
*Cymbopogon nardus* L.	Lemongrass	22.64% geraniol; 7.74% limonene; 7.66% camphene; 6.81% methyl isoeugenol; 5.9% geranyl acetate
*Lavandula officinalis* P.	Lavender	27.11% linalol; 24.4% linalyl acetate; 9.78% β-ocimene; 5.36% caryophyllene; 5.11% 4-terpineol
*Malaeuca alternifolia* Cheel.	Tea Tree	35.88% terpinen-4-ol; 19.65% γ-terpinene; 8.64% α-terpinene; 4.61% p-cymene; 4.07% 1,8-cineole
*Mentha × piperita* Huds var. *officinalis* L.	Mentha of Pancalieri	41.7% menthol; 21.8% menthone; 5.3% 1,8-cineole; 4.8% menthil-acetate; 1.8% limonene
*Pelargonium graveolens* L’Herin.ex.Ait.	Geranium	33.22% citronellal + neral; 5.56% geraniol-formate; 5.71% isomenthone; 4.19% linalol
*Piper nigrum* L.	Black Pepper	50% trans β-carophyllene; 20% limonene; 10% α-pinene; 10% δ-3 carene; 10% β-pinene
*Syzygium aromaticum* L.	Clove bud	78.91% eugenol; 11.64% eugenyl acetate; 6.04% β-caryophyllene; 0.69% α-humulene; 0.27% α-copaene
*Thymus zygis* L.	Thyme	41.18% thymol; 18.99% p-cymene; 5.56% linalool; 5.42% β-carophyllene; 3.6% γ-terpinen
*Zingiber officinali* Roscoe	Ginger	10% β-bisabolene; 10% camphene; 10% β-phellandrene; 5% eucaliptolo; 5% α-pinene

**Table 3:** Minimum inhibitory concentration (MIC) of all the essential oils towards twenty-three *C. auris* isolates, reported as percentage (%) *v*/*v*.

Isolate	GE	TH	LG	TT	CL	CI	MP	LV	PB	CF	BG	GI	LM	MY	MY-N
MOL 1	0.125	0.06	0.06	1	0.06	0.06	0.25	1	>1	>1	>1	>1	>1	>1	>1
MOL 2	0.06	0.06	0.06	0.5	0.06	0.125	0.25	1	>1	>1	>1	>1	>1	>1	>1
MOL 3	0.06	0.06	0.125	1	0.06	0.125	0.5	1	>1	>1	>1	>1	>1	>1	>1
MOL 4	0.06	0.06	0.25	1	0.03	0.06	0.25	1	>1	>1	>1	>1	>1	>1	>1
MOL 5	0.03	0.06	0.06	1	0.06	0.06	0.25	1	>1	>1	>1	>1	>1	>1	>1
MOL 6	0.06	0.06	0.06	0.5	0.06	0.125	0.25	1	>1	>1	>1	>1	>1	>1	>1
MOL 7	0.03	0.06	0.06	1	0.06	0.06	0.125	1	>1	>1	>1	>1	>1	>1	>1
MOL 8	0.03	0.06	0.125	1	0.06	0.125	0.25	1	>1	>1	>1	>1	>1	>1	>1
MOL 9	0.03	0.06	0.06	1	0.03	0.06	0.125	1	>1	>1	>1	>1	>1	>1	>1
MOL 10	0.03	0.03	0.06	1	0.03	0.125	0.125	1	>1	>1	>1	>1	>1	>1	>1
MOL 11	0.125	0.03	0.125	1	0.125	0.125	1	1	>1	>1	>1	>1	>1	>1	>1
MOL 12	0.06	0.03	0.125	1	0.125	0.125	0.5	1	>1	>1	>1	>1	>1	>1	>1
MOL 13	0.03	0.015	0.03	1	0.06	0.06	0.25	1	>1	>1	>1	>1	>1	>1	>1
MOL 14	0.125	0.06	0.125	1	0.125	0.125	0.5	1	>1	>1	>1	>1	>1	>1	>1
TUCC 306	0.06	0.06	0.125	1	0.06	0.06	1	1	>1	>1	>1	>1	>1	>1	>1
TUCC 307	0.06	0.015	0.06	1	0.06	0.03	0.25	1	>1	>1	>1	>1	>1	>1	>1
TUCC 308	0.06	0.03	0.06	1	0.06	0.06	0.25	1	>1	>1	>1	>1	>1	>1	>1
TUCC 309	0.06	0.015	0.06	1	0.06	0.06	0.5	1	>1	>1	>1	>1	>1	>1	>1
TUCC 353	0.03	0.03	0.03	1	0.06	0.06	0.5	1	>1	>1	>1	>1	>1	>1	>1
TUCC 355	0.06	0.03	0.06	1	0.06	0.06	0.25	1	>1	>1	>1	>1	>1	>1	>1
TUCC 356	0.06	0.03	0.06	1	0.06	0.06	0.25	1	>1	>1	>1	>1	>1	>1	>1
TUCC 358	0.06	0.03	0.06	1	0.06	0.03	0.25	1	>1	>1	>1	>1	>1	>1	>1
TUCC 359	0.125	0.03	0.06	1	0.06	0.06	0.25	1	>1	>1	>1	>1	>1	>1	>1

Abbreviations. BG: bergamot; CF: Arabica coffee; CI: cinnamon; CL: clove bud; GE: geranium; GI: ginger; LG: lemongrass; LM: lemon; LV: lavender; MOL: Molinette; MP: mentha of Pancalieri; MY: myrrh; MY-N: Namibian myrrh; PB: black pepper; TH: thyme; TT: tea tree; TUCC: Turin University Culture Collection.

**Table 4:** Minimum fungicidal concentration (MFC) of all the essential oils towards twenty-three *C. auris* isolates, reported as percentage (%) *v*/*v*.

Isolate	GE	TH	LG	TT	CL	CI	MP	LV	PB	CF	BG	GI	LM	MY	MY-N
MOL 1	0.125	0.06	0.125	1	0.125	0.125	0.5	1	>1	>1	>1	>1	>1	>1	>1
MOL 2	0.125	0.5	0.125	1	0.125	0.125	0.5	1	>1	>1	>1	>1	>1	>1	>1
MOL 3	0.125	1	0.125	1	0.125	0.125	0.5	1	>1	>1	>1	>1	>1	>1	>1
MOL 4	0.125	0.125	0.25	1	0.125	0.06	0.25	1	>1	>1	>1	>1	>1	>1	>1
MOL 5	0.06	0.06	0.125	1	0.125	0.06	0.5	1	>1	>1	>1	>1	>1	>1	>1
MOL 6	0.06	0.06	0.125	1	0.125	0.125	0.25	1	>1	>1	>1	>1	>1	>1	>1
MOL 7	0.06	0.06	0.25	1	0.125	0.125	0.5	1	>1	>1	>1	>1	>1	>1	>1
MOL 8	0.03	0.06	0.125	1	0.25	0.125	0.5	1	>1	>1	>1	>1	>1	>1	>1
MOL 9	0.03	0.06	0.125	1	0.06	0.125	1	1	>1	>1	>1	>1	>1	>1	>1
MOL 10	0.06	0.03	0.125	1	0.125	0.25	0.25	1	>1	>1	>1	>1	>1	>1	>1
MOL 11	0.125	0.06	0.25	1	0.25	0.25	1	1	>1	>1	>1	>1	>1	>1	>1
MOL 12	0.125	0.06	0.25	1	0.25	0.25	1	1	>1	>1	>1	>1	>1	>1	>1
MOL 13	0.125	0.06	0.125	1	0.25	0.25	1	1	>1	>1	>1	>1	>1	>1	>1
MOL 14	0.125	0.125	0.125	1	0.25	0.25	1	1	>1	>1	>1	>1	>1	>1	>1
TUCC 306	0.125	0.125	0.25	1	0.125	0.125	1	1	>1	>1	>1	>1	>1	>1	>1
TUCC 307	0.5	0.125	0.5	1	0.125	0.125	>1	1	>1	>1	>1	>1	>1	>1	>1
TUCC 308	0.25	0.125	0.25	1	0.25	0.125	1	1	>1	>1	>1	>1	>1	>1	>1
TUCC 309	0.125	0.06	0.5	1	0.125	0.125	1	1	>1	>1	>1	>1	>1	>1	>1
TUCC 353	0.125	0.125	0.25	1	0.125	0.125	1	1	>1	>1	>1	>1	>1	>1	>1
TUCC 355	0.125	0.06	0.125	1	0.06	0.125	1	1	>1	>1	>1	>1	>1	>1	>1
TUCC 356	0.125	0.06	0.125	1	0.125	0.125	1	1	>1	>1	>1	>1	>1	>1	>1
TUCC 358	0.5	0.06	0.06	1	0.125	0.06	1	1	>1	>1	>1	>1	>1	>1	>1
TUCC 359	0.125	0.0125	0.5	1	0.125	0.125	1	1	>1	>1	>1	>1	>1	>1	>1

Abbreviations. BG: bergamot; CF: Arabica coffee; CI: cinnamon; CL: clove bud; GE: geranium; GI: ginger; LG: lemongrass; LM: lemon; LV: lavender; MOL: Molinette; MP: mentha of Pancalieri; MY: myrrh; MY-N: Namibian myrrh; PB: black pepper; TH: thyme; TT: tea tree; TUCC: Turin University Culture Collection.

**Table 5:** Inhibition halo diameters (mm) of fluconazole, micafungin, caspofungin and 5-flucytosine towards *C. auris* MOL 10 and MOL 11, by agar disc diffusion assay.

Strain	FLU (1000/1 µg/mL)	MYC (5/1 µg/mL)	CAS (5/1 µg/mL)	5-FL (5/1 µg/mL)
MOL 10	0	24.05 ± 0.01	14.52 ± 1.46	39.21 ± 1.62
MOL 11	0	24.11 ± 0.01	13.52 ± 0.01	39.72 ± 0.5

Abbreviations. 5-FL: 5-flucytosin; CAS: caspofungin; FLU: fluconazole; MOL: Molinette; MYC: micafungin.

**Table 6:** Inhibition halo diameters (mm) of thyme, clove bud, lemongrass, cinnamon, geranium and mentha of Pancalieri towards *C. auris* MOL 10 and MOL 11, by agar disc diffusion assay.

Strain	Concentration	TH	CL	LG	CI	GE	MP
MOL 10	25%	40.94 ± 0.65	32.03 ± 0.49	23.63 ± 1.41	15.38 ± 1.39	10.55 ± 0.85	9.60 ± 0.49
	75%	59.57 ± 0.55	35.01 ± 0.03	35.58 ± 1.22	19.05 ± 0.90	19.08 ± 0.63	12.08 ± 0.03
	100%	63.88 ± 0.66	36.77 ± 0.02	36.87 ± 0.28	35.84 ± 1.65	26.04 ± 0.59	14.10 ± 0.57
MOL 11	25%	50.77 ± 0.80	32.35 ± 0.40	27.5 ± 1.04	15.92 ± 1.41	12.49 ± 1.18	9.95 ± 0.26
	75%	56.82 ± 0.75	36.09 ± 0.51	30.47 ± 1.11	19.86 ± 0.71	16.32 ± 1.71	14.15 ± 0.45
	100%	63.69 ± 0.77	39.42 ± 0.23	31.46 ± 1.31	32.82 ± 0.68	20.43 ± 1.11	19.53 ± 1.11

Abbreviations. CI: cinnamon; CL: clove bud; GE: geranium; LG: lemongrass; MOL: Molinette; MP: mentha of Pancalieri; TH: thyme.

**Table 7:** Interaction among the four traditional antifungals and the six selected EOs against *C. auris* MOL 10.

Antifungal	Essential Oil	FICI	Interpretation
Fluconazole	Geranium	0.98	Additive
Fluconazole	Thyme	2.5	Indifferent
Fluconazole	Lemongrass	0.99	Additive
Fluconazole	Clove bud	1.06	Indifferent
Fluconazole	Cinnamon	0.98	Additive
Fluconazole	Mentha of Pancalieri	0.75	Additive
Micafungin	Geranium	0.5	Synergic
Micafungin	Thyme	0.5	Synergic
Micafungin	Lemongrass	0.75	Additive
Micafungin	Clove bud	1.06	Indifferent
Micafungin	Cinnamon	0.5	Synergic
Micafungin	Mentha of Pancalieri	0.99	Additive
Caspofungin	Geranium	1.06	Indifferent
Caspofungin	Thyme	8.36	Antagonistic
Caspofungin	Lemongrass	0.75	Additive
Caspofungin	Clove bud	0.97	Additive
Caspofungin	Cinnamon	1.25	Indifferent
Caspofungin	Mentha of Pancalieri	0.98	Additive
5-Flucytosine	Geranium	0.97	Additive
5-Flucytosine	Thyme	1.5	Indifferent
5-Flucytosine	Lemongrass	0.98	Additive
5-Flucytosine	Clove bud	0.97	Additive
5-Flucytosine	Cinnamon	1.5	Indifferent
5-Flucytosine	Mentha of Pancalieri	1.5	Indifferent

Interpretation colours. Green: synergic; yellow: additive; orange: indifferent; red: antagonistic.

**Table 8:** Interaction among the four traditional antifungals and the six selected EOs against *C. auris* MOL 11.

Antifungal	Essential Oil	FICI	Interpretation
Fluconazole	Geranium	0.98	Additive
Fluconazole	Thyme	2	Indifferent
Fluconazole	Lemongrass	2	Indifferent
Fluconazole	Clove bud	0.97	Additive
Fluconazole	Cinnamon	1.5	Indifferent
Fluconazole	Mentha of Pancalieri	0.5	Synergic
Micafungin	Geranium	2	Indifferent
Micafungin	Thyme	0.98	Additive
Micafungin	Lemongrass	0.5	Synergic
Micafungin	Clove bud	0.5	Synergic
Micafungin	Cinnamon	0.5	Synergic
Micafungin	Mentha of Pancalieri	2	Indifferent
Caspofungin	Geranium	0.98	Additive
Caspofungin	Thyme	2	Indifferent
Caspofungin	Lemongrass	0.99	Additive
Caspofungin	Clove bud	0.97	Additive
Caspofungin	Cinnamon	0.97	Additive
Caspofungin	Mentha of Pancalieri	0.97	Additive
5-Flucytosine	Geranium	0.97	Additive
5-Flucytosine	Thyme	2	Indifferent
5-Flucytosine	Lemongrass	1.5	Indifferent
5-Flucytosine	Clove bud	0.98	Additive
5-Flucytosine	Cinnamon	1.5	Indifferent
5-Flucytosine	Mentha of Pancalieri	0.5	Synergic

Interpretation colours. Green: synergic; yellow: additive; orange: indifferent.

## Data Availability

The original contributions presented in the study are included in the article; further inquiries can be directed to the corresponding author.

## References

[1] Jangir P., Kalra S., Tanwar S., Bari V.K. (2023). Azole Resistance in *Candida auris*: Mechanisms and Combinatorial Therapy. APMIS.

[2] Pekard-Amenitsch S., Schriebl A., Posawetz W., Willinger B., Kölli B., Buzina W. (2018). Isolation of *Candida auris* from Ear of Otherwise Healthy Patient, Austria, 2018. Emerg. Infect. Dis..

[3] Satoh K., Makimura K., Hasumi Y., Nishiyama Y., Uchida K., Yamaguchi H. (2009). *Candida auris* sp. Nov., a Novel Ascomycetous Yeast Isolated from the External Ear Canal of an Inpatient in a Japanese Hospital. Microbiol. Immunol..

[4] Tsay S., Kallen A., Jackson B.R., Chiller T.M., Vallabhaneni S. (2018). Approach to the Investigation and Management of Patients With *Candida auris*, an Emerging Multidrug-Resistant Yeast. Clin. Infect. Dis..

[5] Noble B.A., Jurcic Smith K.L., Jones J.D., Galvin B.W., Timbrook T.T. (2023). *Candida auris* Rates in Blood Culture on the Rise: Results of US Surveillance. Microbiol. Spectr..

[6] Geremia N., Brugnaro P., Solinas M., Scarparo C., Panese S. (2023). *Candida auris* as an Emergent Public Health Problem: A Current Update on European Outbreaks and Cases. Healthcare.

[7] Lyman M., Forsberg K., Sexton D.J., Chow N.A., Lockhart S.R., Jackson B.R., Chiller T. (2023). Worsening Spread of *Candida auris* in the United States, 2019 to 2021. Ann. Intern. Med..

[8] Bravo Ruiz G., Lorenz A. (2021). What Do We Know about the Biology of the Emerging Fungal Pathogen of Humans *Candida auris*?. Microbiol. Res..

[9] Chowdhary A., Voss A., Meis J.F. (2016). Multidrug-Resistant *Candida auris*: “new Kid on the Block” in Hospital-Associated Infections?. J. Hosp. Infect..

[10] Cortegiani A., Misseri G., Fasciana T., Giammanco A., Giarratano A., Chowdhary A. (2018). Epidemiology, Clinical Characteristics, Resistance, and Treatment of Infections by *Candida auris*. J. Intensive Care.

[11] Osei Sekyere J. (2018). *Candida auris*: A Systematic Review and Meta-Analysis of Current Updates on an Emerging Multidrug-Resistant Pathogen. Microbiologyopen.

[12] Casadevall A., Kontoyiannis D.P., Robert V. (2019). On the Emergence of *Candida auris*: Climate Change, Azoles, Swamps, and Birds. mBio.

[13] Wang X., Bing J., Zheng Q., Zhang F., Liu J., Yue H., Tao L., Du H., Wang Y., Wang H. (2018). The First Isolate of *Candida auris* in China: Clinical and Biological Aspects. Emerg. Microbes Infect..

[14] Singh R., Kaur M., Chakrabarti A., Shankarnarayan S.A., Rudramurthy S.M. (2019). Biofilm Formation by *Candida auris* Isolated from Colonising Sites and Candidemia Cases. Mycoses.

[15] Lockhart S.R., Etienne K.A., Vallabhaneni S., Farooqi J., Chowdhary A., Govender N.P., Colombo A.L., Calvo B., Cuomo C.A., Desjardins C.A. (2017). Simultaneous Emergence of Multidrug-Resistant *Candida auris* on 3 Continents Confirmed by Whole-Genome Sequencing and Epidemiological Analyses. Clin. Infect. Dis..

[16] Fernandes L., Ribeiro R., Costa R., Henriques M., Rodrigues M.E. (2022). Essential Oils as a Good Weapon against Drug-Resistant *Candida auris*. Antibiotics.

[17] Di Vito M., Garzoli S., Rosato R., Mariotti M., Gervasoni J., Santucci L., Ovidi E., Cacaci M., Lombarini G., Torelli R. (2023). A New Potential Resource in the Fight against *Candida auris*: The Cinnamomum Zeylanicum Essential Oil in Synergy with Antifungal Drug. Microbiol. Spectr..

[18] Ostrowsky B., Greenko J., Adams E., Quinn M., O’Brien B., Chaturvedi V., Berkow E., Vallabhaneni S., Forsberg K., Chaturvedi S. (2020). *Candida auris* Isolates Resistant to Three Classes of Antifungal Medications—New York, 2019. MMWR Morb. Mortal. Wkly. Rep..

[19] Revie N.M., Iyer K.R., Robbins N., Cowen L.E. (2018). Antifungal Drug Resistance: Evolution, Mechanisms and Impact. Curr. Opin. Microbiol..

[20] Rajeshkumar R., Sundararaman M. (2012). Emergence of *Candida* spp. and Exploration of Natural Bioactive Molecules for Anticandidal Therapy—Status Quo. Mycoses.

[21] Mandras N., Roana J., Scalas D., Del Re S., Cavallo L., Ghisetti V., Tullio V. (2021). The Inhibition of Non-Albicans *Candida* Species and Uncommon Yeast Pathogens by Selected Essential Oils and Their Major Compounds. Molecules.

[22] Allizond V., Cavallo L., Roana J., Mandras N., Cuffini A.M., Tullio V., Banche G. (2023). In Vitro Antifungal Activity of Selected Essential Oils against Drug-Resistant Clinical *Aspergillus* Spp. Strains. Molecules.

[23] Cavaleiro C., Pinto E., Gonçalves M.J., Salgueiro L. (2006). Antifungal Activity of *Juniperus* Essential Oils against Dermatophyte, *Aspergillus* and *Candida* Strains. J. Appl. Microbiol..

[24] Tisserand R., Young R. (2013). Essential Oil Safety: A Guide for Health Care Professionals.

[25] Comini S., Scutera S., Sparti R., Banche G., Coppola B., Bertea C.M., Bianco G., Gatti N., Cuffini A.M., Palmero P. (2022). Combination of Poly(ε-caprolactone) Biomaterials and Essential Oils to Achieve Anti-Bacterial and Osteo-Proliferative Properties for 3D-Scaffolds in Regenerative Medicine. Pharmaceutics.

[26] Adukwu E.C., Bowles M., Edwards-Jones V., Bone H. (2016). Antimicrobial Activity, Cytotoxicity and Chemical Analysis of Lemongrass Essential Oil (*Cymbopogon flexuosus*) and Pure Citral. Appl. Microbiol. Biotechnol..

[27] Palmeira-de-Oliveira A., Salgueiro L., Palmeira-de-Oliveira R., Martinez-de-Oliveira J., Pina-Vaz C., Queiroz J.A., Rodrigues A.G. (2009). Anti-Candida Activity of Essential Oils. Mini Rev. Med. Chem..

[28] Swamy M.K., Akhtar M.S., Sinniah U.R. (2016). Antimicrobial Properties of Plant Essential Oils against Human Pathogens and Their Mode of Action: An Updated Review. Evid.-Based Complement. Alternat Med..

[29] Tran H.N.H., Graham L., Adukwu E.C. (2020). In Vitro Antifungal Activity of *Cinnamomum zeylanicum* Bark and Leaf Essential Oils against *Candida albicans* and *Candida auris*. Appl. Microbiol. Biotechnol..

[30] Mukherjee P.K., Sheehan D.J., Hitchcock C.A., Ghannoum M.A. (2005). Combination Treatment of Invasive Fungal Infections. Clin. Microbiol. Rev..

[31] Stringaro A., Vavala E., Colone M., Pepi F., Mignogna G., Garzoli S., Cecchetti S., Ragno R., Angiolella L. (2014). Effects of *Mentha suaveolens* Essential Oil Alone or in Combination with Other Drugs in *Candida albicans*. Evid.-Based Complement. Alternat Med..

[32] Silva F., Ferreira S., Duarte A., Mendonça D.I., Domingues F.C. (2011). Antifungal Activity of *Coriandrum sativum* Essential Oil, Its Mode of Action against *Candida* Species and Potential Synergism with Amphotericin B. Phytomedicine.

[33] Parker R.A., Gabriel K.T., Graham K.D., Butts B.K., Cornelison C.T. (2022). Antifungal Activity of Select Essential Oils against *Candida auris* and Their Interactions with Antifungal Drugs. Pathogens.

[34] Bidaud A.-L., Schwarz P., Herbreteau G., Dannaoui E. (2021). Techniques for the Assessment of In Vitro and In Vivo Antifungal Combinations. J. Fungi.

[35] O’Brien B., Chaturvedi S., Chaturvedi V. (2020). In Vitro Evaluation of Antifungal Drug Combinations against Multidrug-Resistant *Candida auris* Isolates from New York Outbreak. Antimicrob. Agents Chemother..

[36] Billamboz M., Fatima Z., Hameed S., Jawhara S. (2021). Promising Drug Candidates and New Strategies for Fighting against the Emerging Superbug *Candida auris*. Microorganisms.

[37] Jacobs S.E., Jacobs J.L., Dennis E.K., Taimur S., Rana M., Patel D., Gitman M., Patel G., Schaefer S., Iyer K. (2022). *Candida auris* Pan-Drug-Resistant to Four Classes of Antifungal Agents. Antimicrob. Agents Chemother..

[38] Fakhim H., Chowdhary A., Prakash A., Vaezi A., Dannaoui E., Meis J.F., Badali H. (2017). In Vitro Interactions of Echinocandins with Triazoles against Multidrug-Resistant *Candida auris*. Antimicrob. Agents Chemother..

[39] Shaban S., Patel M., Ahmad A. (2020). Improved Efficacy of Antifungal Drugs in Combination with Monoterpene Phenols against *Candida auris*. Sci. Rep..

[40] Rosato R., Napoli E., Granata G., Di Vito M., Garzoli S., Geraci C., Rizzo S., Torelli R., Sanguinetti M., Bugli F. (2023). Study of the Chemical Profile and Anti-Fungal Activity against *Candida auris* of Cinnamomum Cassia Essential Oil and of Its Nano-Formulations Based on Polycaprolactone. Plants.

[41] Di Vito M., Rosato R., Rizzo S., Cacaci M., Urbani A., Sanguinettii M., Bugli F. (2024). Enhancing Fluconazole Reactivation against *Candida auris*: Efficacy of Cinnamomum Zeylanicum Essential Oil versus Cinnamaldehyde. Microbiol. Spectr..

[42] Bravo-Chaucanés C.P., Vargas-Casanova Y., Chitiva-Chitiva L.C., Ceballos-Garzon A., Modesti-Costa G., Parra-Giraldo C.M. (2022). Evaluation of Anti-Candida Potential of Piper Nigrum Extract in Inhibiting Growth, Yeast-Hyphal Transition, Virulent Enzymes, and Biofilm Formation. J. Fungi.

[43] Tullio V., Mandras N., Allizond V., Nostro A., Roana J., Merlino C., Banche G., Scalas D., Cuffini A.M. (2012). Positive Interaction of Thyme (Red) Essential Oil with Human Polymorphonuclear Granulocytes in Eradicating Intracellular *Candida albicans*. Planta Med..

[44] Cao C., Wei D., Xu L., Hu J., Qi J., Zhou Y. (2021). Characterization of Tea Tree Essential Oil and Large-Ring Cyclodextrins (CD9–CD22) Inclusion Complex and Evaluation of Its Thermal Stability and Volatility. J. Sci. Food Agric..

[45] Ruiz-Duran J., Torres R., Stashenko E.E., Ortiz C. (2023). Antifungal and Antibiofilm Activity of Colombian Essential Oils against Different *Candida* Strains. Antibiotics.

[46] Zapata-Zapata C., Loaiza-Oliva M., Martínez-Pabón M.C., Stashenko E.E., Mesa-Arango A.C. (2022). In Vitro Activity of Essential Oils Distilled from Colombian Plants against *Candidaauris* and Other Candida Species with Different Antifungal Susceptibility Profiles. Molecules.

[47] Maione A., La Pietra A., de Alteriis E., Mileo A., De Falco M., Guida M., Galdiero E. (2022). Effect of Myrtenol and Its Synergistic Interactions with Antimicrobial Drugs in the Inhibition of Single and Mixed Biofilms of *Candida auris* and *Klebsiella pneumoniae*. Microorganisms.

[48] Kim H.-R., Eom Y.-B. (2021). Antifungal and Anti-biofilm Effects of 6-shogaol against *Candida auris*. J. Appl. Microbiol..

[49] Carrasco H., Raimondi M., Svetaz L., Liberto M.D., Rodriguez M.V., Espinoza L., Madrid A., Zacchino S. (2012). Antifungal Activity of Eugenol Analogues. Influence of Different Substituents and Studies on Mechanism of Action. Molecules.

[50] Chami N., Bennis S., Chami F., Aboussekhra A., Remmal A. (2005). Study of Anticandidal Activity of Carvacrol and Eugenol in Vitro and in Vivo. Oral Microbiol. Immunol..

[51] Shahina Z., Ndlovu E., Persaud O., Sultana T., Dahms T.E.S. (2022). *Candida albicans* Reactive Oxygen Species (ROS)-Dependent Lethality and ROS-Independent Hyphal and Biofilm Inhibition by Eugenol and Citral. Microbiol. Spectr..

[52] Bidaud A.L., Djenontin E., Botterel F., Chowdhary A., Dannaoui E. (2020). Colistin Interacts Synergistically with Echinocandins against *Candida auris*. Int. J. Antimicrob. Agents.

[53] Halliday C., Kim H.Y., Tay E., Chen S.C.A., Alffenaar J.-W. (2023). Exploring Synergy between Azole Antifungal Drugs and Statins for *Candida auris*. J. Antimicrob. Chemother..

[54] Davis H.R., Ashcraft D.S., Pankey G.A. (2021). In Vitro Interaction of Fluconazole and Trimethoprim-Sulfamethoxazole against *Candida auris* Using ETEST and Checkerboard Methods. J. Investig. Med..

[55] Menotti F., Scutera S., Coppola B., Longo F., Mandras N., Cavallo L., Comini S., Sparti R., Fiume E., Cuffini A.M. (2023). Tuning of Silver Content on the Antibacterial and Biological Properties of Poly(ɛ-caprolactone)/Biphasic Calcium Phosphate 3D-Scaffolds for Bone Tissue Engineering. Polymers.

[56] Puiu R.A., Bîrcă A.C., Grumezescu V., Duta L., Oprea O.C., Holban A.M., Hudiță A., Gălățeanu B., Balaure P.C., Grumezescu A.M. (2023). Multifunctional Polymeric Biodegradable and Biocompatible Coatings Based on Silver Nanoparticles: A Comparative In Vitro Study on Their Cytotoxicity towards Cancer and Normal Cell Lines of Cytostatic Drugs versus Essential-Oil-Loaded Nanoparticles and on Their Antimicrobial and Antibiofilm Activities. Pharmaceutics.

